# (5-Benzoyl-2-methyl-4-{[1-(pyridin-4-yl)-1*H*-1,2,3-triazol-4-yl]meth­oxy}-1-benzofuran-7-yl)(phen­yl)methanone

**DOI:** 10.1107/S1600536812023720

**Published:** 2012-05-31

**Authors:** Xiao-qin Zhang, Hai-Liang Zhang, Zhu-Yong Dong, Qiang Qian, Yu-Guang Wang

**Affiliations:** a College of Biological and Environmental Engineering, Zhejiang University of Technology, Hangzhou 310014, People’s Republic of China; b Zhejiang SiXian Pharmaceutical Co. Ltd, ShaoXing 312065, People’s Republic of China

## Abstract

The crystal structure of the title compound, C_31_H_22_N_4_O_4_, features weak C—H⋯O inter­actions. The dihedral angle between the fused benzene and furan rings is 2.49 (15)°, while that between the triazole and pyridine rings is 10.23(18)°.

## Related literature

For bioactive nnitro­gen-linked heterocyclic compounds, see: Anderson *et al.* (2004 ▶); Ha *et al.* (2009 ▶); Liu *et al.* (2011 ▶); Tan *et al.* (2012 ▶); Venkatesan *et al.* (2010 ▶); Yim *et al.* (2010 ▶). For the bioactivity of benzofuran analogues substituted by heterocyclic moieties, see: El-Shehry *et al.* (2010 ▶); Kaldrikyan *et al.* (2009 ▶); Saberi *et al.* (2006 ▶). For a related structure, see: Liu *et al.* (2012 ▶).
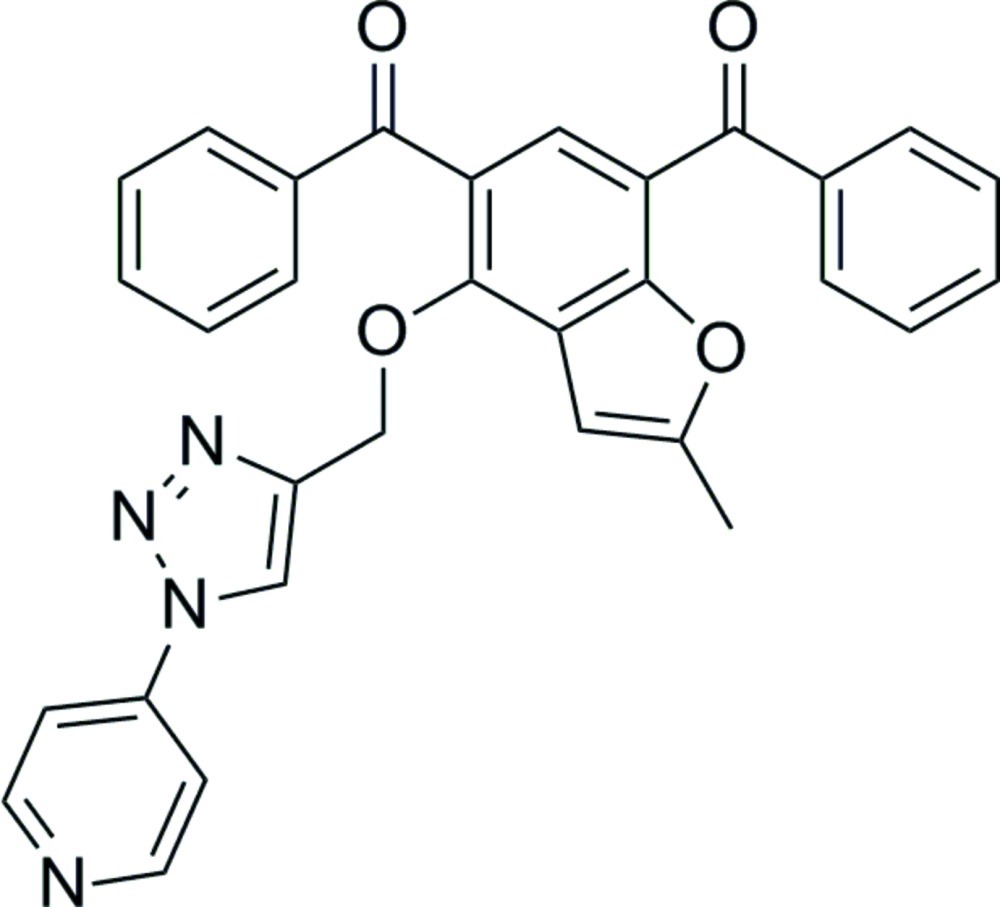


## Experimental

### 

#### Crystal data

C_31_H_22_N_4_O_4_
*M* *_r_* = 514.53
Triclinic, 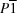

*a* = 10.11 (2) Å
*b* = 10.87 (3) Å
*c* = 11.64 (3) Å
α = 94.73 (4)°
β = 92.07 (3)°
γ = 92.05 (4)°
*V* = 1273 (5) Å^3^
*Z* = 2
Mo *K*α radiation
μ = 0.09 mm^−1^
*T* = 296 K
0.38 × 0.22 × 0.13 mm

#### Data collection

Bruker SMART CCD area-detector diffractometer
Absorption correction: multi-scan (*SADABS*; Sheldrick, 1996 ▶) *T*_min_ = 0.966, *T*_max_ = 0.988
9629 measured reflections
4699 independent reflections
2743 reflections with *I* > 2σ(*I*)
*R*_int_ = 0.044

#### Refinement

*R*[*F* ^2^ > 2σ(*F* ^2^)] = 0.053
*wR*(*F* ^2^) = 0.200
*S* = 0.85
4699 reflections
353 parameters
H-atom parameters constrained
Δρ_max_ = 0.26 e Å^−3^
Δρ_min_ = −0.17 e Å^−3^

### 

Data collection: *SMART* (Bruker, 2004 ▶); cell refinement: *SAINT* (Bruker, 2004 ▶); data reduction: *SAINT*; program(s) used to solve structure: *SHELXTL* (Sheldrick, 2008 ▶); program(s) used to refine structure: *SHELXTL*; molecular graphics: *SHELXTL*; software used to prepare material for publication: *SHELXTL*.

## Supplementary Material

Crystal structure: contains datablock(s) global, I. DOI: 10.1107/S1600536812023720/ds2193sup1.cif

Structure factors: contains datablock(s) I. DOI: 10.1107/S1600536812023720/ds2193Isup2.hkl

Supplementary material file. DOI: 10.1107/S1600536812023720/ds2193Isup3.cml

Additional supplementary materials:  crystallographic information; 3D view; checkCIF report

## Figures and Tables

**Table 1:** Hydrogen-bond geometry (Å, °)

*D*—H⋯*A*	*D*—H	H⋯*A*	*D*⋯*A*	*D*—H⋯*A*
C11—H11⋯O3^i^	0.93	2.33	3.225 (10)	161
C16—H16⋯O3^i^	0.93	2.55	3.473 (10)	171

Symmetry code: (i) 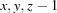.

**Fig. 1.**
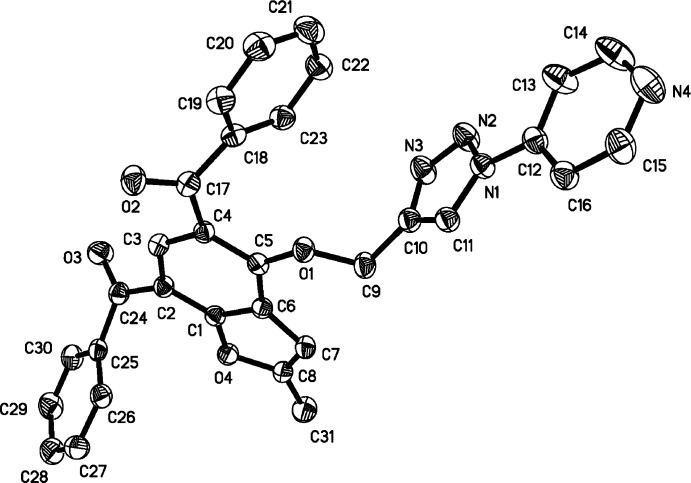
View of the title compound showing the atom numbering scheme and the ellipsoids at the 50% probability level.

## supplementary crystallographic information

### Experimental

General procedure to synthesize the title compound: Under a positive pressure of nitrogen, to a suspension of the swollen 2-polystyrene supported selanylmethyl-4-(prop-2-ynyloxy)-5,7-dibenzoyl- *2,3*-dihydro-benzofuran in DMSO (30 mL) was added the mixed solution of 0.2 g (4.0 mmol) Cu_2_SO_4_ 5H_2_O and 0.8 g(4.5 mmol) ascorbic acid in 10 mL water, 5.0 mmol 4-Azido-pyridine. After stirring for 15 h at 60°C, the resin was collected by filtration, washed with H_2_O (30 mL×2), THF (20 mL×1), hot DMF (15 mL×1), H2O (30 mL×1), THF (20 mL×1), THF/H_2_O (2:1) (20 mL×2), hot DMF (15 mL×1), THF (20 mL×1), THF/H_2_O (2:1) (20 mL×2), THF (20 mL×2). The washed resin was suspended in THF (15 mL), 30% H_2_O_2_ (20.0 equiv) was added, and the mixture was stirred for 10 h at room temperature. The resin was collected by filtration, washed with H_2_O (20 mL×2), THF (10 mL×2), THF/H_2_O (2:1) (10 mL×2), THF (10 mL×2) , CH_2_Cl_2_ (10 mL×2), toluene (10 mL×2). The washed resin was suspended in 15 mL toluene, DBU (0.4 equiv) was added, and the mixture was stirred for 5.0 h at 80°C. The mixture was filtered, and the resin was washed with CH_2_Cl_2_ (15 mL ×2). The filtrate was washed with 0.25M HCl (30 mL×2), saturated sodium bicarbonate solution(35 mL×2), dried with anhydrous magnesium sulfate, and evaporated to dryness under vacuum to obtain the title compound. Further purification was via flash chromatography with n-hexanes-EtOAc (3:1, V/V) as the eluent for microanalyses. ^1^H-NMR(CDCl_3_, 400MHz, Bruker Avance spectrometer): δ 8.78-8.76 (d, 2H, J=5.6Hz), 7.85-7.81 (m, 4H), 7.64-7.40 (m, 10H), 6.77 (d, 1H, J=0.8Hz), 5.52 (s, 2H), 2.47 (s, 3H); 13C-NMR(CDCl3): δ 195.6, 192,8, 157.7, 156.2, 152.5, 151.9, 145.4, 143.1, 138.4, 137.9, 133.4, 133.2, 130.3, 130.2, 128.7, 128.6, 128.1, 125.0, 122.1, 120.5, 118.3, 113.9, 101.2, 67.1, 14.3; MS(ESI) m/z 515 (M+H)^+^. The title compound was recrystallized from CHCl_2_ at room temperature to give the desired crystals suitable for single-crystal X-ray diffraction.

### Refinement

All H atoms were positioned geometrically and constrained to ride on their parent atoms (N—H = 0.86 Å and U_iso_(H) = 1.2U_eq_(N); C—H = 0.93 and 0.97 Å for aromatic and methylene H atoms with U_iso_(H) = 1.2U_eq_(C), respectively.

### Crystal data

**Table:**

C_31_H_22_N_4_O_4_	*Z* = 2
*M**_r_* = 514.53	*F*(000) = 536
Triclinic, *P*1	*D*_x_ = 1.343 Mg m^−^^3^
Hall symbol: -P 1	Mo *K*α radiation, λ = 0.71073 Å
*a* = 10.11 (2) Å	Cell parameters from 1346 reflections
*b* = 10.87 (3) Å	θ = 2.5–21.0°
*c* = 11.64 (3) Å	µ = 0.09 mm^−^^1^
α = 94.73 (4)°	*T* = 296 K
β = 92.07 (3)°	Block, colourless
γ = 92.05 (4)°	0.38 × 0.22 × 0.13 mm
*V* = 1273 (5) Å^3^	

### Data collection

**Table:**

Bruker SMART CCD area-detector diffractometer	4699 independent reflections
Radiation source: fine-focus sealed tube	2743 reflections with *I* > 2σ(*I*)
Graphite monochromator	*R*_int_ = 0.044
phi and ω scans	θ_max_ = 25.5°, θ_min_ = 2.5°
Absorption correction: multi-scan (*SADABS*; Sheldrick, 1996)	*h* = −12→12
*T*_min_ = 0.966, *T*_max_ = 0.988	*k* = −12→13
9629 measured reflections	*l* = −14→14

### Refinement

**Table:**

Refinement on *F*^2^	Primary atom site location: structure-invariant direct methods
Least-squares matrix: full	Secondary atom site location: difference Fourier map
*R*[*F*^2^ > 2σ(*F*^2^)] = 0.053	Hydrogen site location: inferred from neighbouring sites
*wR*(*F*^2^) = 0.200	H-atom parameters constrained
*S* = 0.85	*w* = 1/[σ^2^(*F*_o_^2^) + (0.1*P*)^2^ + 0.4387*P*] where *P* = (*F*_o_^2^ + 2*F*_c_^2^)/3
4699 reflections	(Δ/σ)_max_ < 0.001
353 parameters	Δρ_max_ = 0.26 e Å^−^^3^
0 restraints	Δρ_min_ = −0.17 e Å^−^^3^

### Special details

**Table:**

Geometry. All esds (except the esd in the dihedral angle between two l.s. planes) are estimated using the full covariance matrix. The cell esds are taken into account individually in the estimation of esds in distances, angles and torsion angles; correlations between esds in cell parameters are only used when they are defined by crystal symmetry. An approximate (isotropic) treatment of cell esds is used for estimating esds involving l.s. planes.
Refinement. Refinement of F^2^ against ALL reflections. The weighted R-factor wR and goodness of fit S are based on F^2^, conventional R-factors R are based on F, with F set to zero for negative F^2^. The threshold expression of F^2^ > 2sigma(F^2^) is used only for calculating R-factors(gt) etc. and is not relevant to the choice of reflections for refinement. R-factors based on F^2^ are statistically about twice as large as those based on F, and R- factors based on ALL data will be even larger.

### Fractional atomic coordinates and isotropic or equivalent isotropic displacement parameters (Å^2^)

**Table:**

	*x*	*y*	*z*	*U*_iso_*/*U*_eq_	
N1	0.4387 (2)	−0.1220 (2)	−0.3567 (2)	0.0473 (6)	
N2	0.5280 (3)	−0.1333 (3)	−0.2672 (2)	0.0622 (8)	
N3	0.4837 (3)	−0.0696 (3)	−0.1759 (2)	0.0614 (8)	
N4	0.5183 (3)	−0.2859 (3)	−0.6907 (3)	0.0806 (10)	
O1	0.20278 (18)	−0.01035 (18)	−0.05837 (16)	0.0467 (5)	
O2	−0.0057 (2)	−0.2440 (2)	0.12502 (19)	0.0610 (6)	
O3	0.1408 (2)	0.0551 (2)	0.48995 (19)	0.0654 (7)	
O4	0.19574 (18)	0.30077 (17)	0.25525 (16)	0.0464 (5)	
C1	0.1656 (3)	0.1786 (3)	0.2199 (2)	0.0406 (7)	
C2	0.1172 (3)	0.0928 (3)	0.2923 (2)	0.0425 (7)	
C3	0.0985 (3)	−0.0274 (3)	0.2403 (2)	0.0448 (7)	
H3	0.0657	−0.0882	0.2846	0.054*	
C4	0.1268 (2)	−0.0616 (3)	0.1240 (2)	0.0405 (7)	
C5	0.1774 (3)	0.0282 (3)	0.0540 (2)	0.0398 (7)	
C6	0.1954 (2)	0.1526 (3)	0.1030 (2)	0.0401 (7)	
C7	0.2451 (3)	0.2700 (3)	0.0659 (2)	0.0448 (7)	
H7	0.2711	0.2844	−0.0078	0.054*	
C8	0.2462 (3)	0.3540 (3)	0.1584 (2)	0.0452 (7)	
C9	0.2956 (3)	0.0639 (3)	−0.1195 (3)	0.0516 (8)	
H9A	0.3597	0.1067	−0.0648	0.062*	
H9B	0.2482	0.1253	−0.1586	0.062*	
C10	0.3658 (3)	−0.0175 (3)	−0.2056 (2)	0.0465 (7)	
C11	0.3382 (3)	−0.0494 (3)	−0.3205 (3)	0.0474 (7)	
H11	0.2660	−0.0265	−0.3646	0.057*	
C12	0.4637 (3)	−0.1773 (3)	−0.4698 (3)	0.0491 (7)	
C13	0.5661 (4)	−0.2603 (4)	−0.4842 (3)	0.0738 (11)	
H13	0.6175	−0.2813	−0.4214	0.089*	
C14	0.5883 (4)	−0.3104 (4)	−0.5955 (4)	0.0874 (13)	
H14	0.6567	−0.3648	−0.6046	0.105*	
C15	0.4212 (4)	−0.2071 (4)	−0.6724 (3)	0.0698 (10)	
H15	0.3701	−0.1886	−0.7363	0.084*	
C16	0.3902 (3)	−0.1506 (3)	−0.5659 (3)	0.0567 (8)	
H16	0.3216	−0.0960	−0.5596	0.068*	
C17	0.0912 (3)	−0.1942 (3)	0.0833 (3)	0.0468 (7)	
C18	0.1739 (3)	−0.2720 (3)	0.0022 (2)	0.0448 (7)	
C19	0.1132 (3)	−0.3795 (3)	−0.0558 (3)	0.0547 (8)	
H19	0.0227	−0.3952	−0.0504	0.066*	
C20	0.1884 (4)	−0.4627 (3)	−0.1214 (3)	0.0639 (9)	
H20	0.1477	−0.5333	−0.1595	0.077*	
C21	0.3240 (4)	−0.4402 (3)	−0.1299 (3)	0.0683 (10)	
H21	0.3739	−0.4959	−0.1733	0.082*	
C22	0.3846 (3)	−0.3340 (3)	−0.0732 (3)	0.0627 (9)	
H22	0.4752	−0.3188	−0.0788	0.075*	
C23	0.3098 (3)	−0.2500 (3)	−0.0078 (3)	0.0520 (8)	
H23	0.3510	−0.1789	0.0293	0.062*	
C24	0.0941 (3)	0.1209 (3)	0.4186 (2)	0.0456 (7)	
C25	0.0118 (3)	0.2283 (3)	0.4561 (2)	0.0447 (7)	
C26	−0.0814 (3)	0.2755 (3)	0.3805 (3)	0.0522 (8)	
H26	−0.0883	0.2445	0.3035	0.063*	
C27	−0.1641 (4)	0.3690 (3)	0.4208 (3)	0.0676 (10)	
H27	−0.2274	0.3984	0.3710	0.081*	
C28	−0.1517 (4)	0.4178 (3)	0.5349 (3)	0.0737 (11)	
H28	−0.2062	0.4803	0.5614	0.088*	
C29	−0.0573 (4)	0.3730 (3)	0.6102 (3)	0.0698 (10)	
H29	−0.0478	0.4067	0.6863	0.084*	
C30	0.0217 (3)	0.2784 (3)	0.5712 (3)	0.0571 (8)	
H30	0.0825	0.2474	0.6221	0.068*	
C31	0.2916 (4)	0.4852 (3)	0.1780 (3)	0.0660 (10)	
H31A	0.3324	0.5105	0.1101	0.099*	
H31B	0.3547	0.4950	0.2421	0.099*	
H31C	0.2171	0.5351	0.1946	0.099*	

### Atomic displacement parameters (Å^2^)

**Table:**

	*U*^11^	*U*^22^	*U*^33^	*U*^12^	*U*^13^	*U*^23^
N1	0.0469 (13)	0.0519 (15)	0.0426 (14)	0.0057 (11)	0.0023 (11)	−0.0015 (12)
N2	0.0580 (16)	0.087 (2)	0.0416 (15)	0.0196 (15)	−0.0011 (12)	−0.0016 (14)
N3	0.0566 (16)	0.083 (2)	0.0443 (16)	0.0135 (14)	0.0016 (12)	−0.0016 (14)
N4	0.080 (2)	0.090 (2)	0.066 (2)	0.0052 (18)	0.0064 (17)	−0.0285 (18)
O1	0.0542 (12)	0.0487 (12)	0.0367 (11)	−0.0012 (9)	0.0065 (9)	0.0000 (9)
O2	0.0587 (13)	0.0587 (14)	0.0641 (15)	−0.0120 (11)	0.0105 (11)	−0.0002 (11)
O3	0.0742 (15)	0.0765 (16)	0.0481 (14)	0.0220 (12)	0.0009 (11)	0.0133 (12)
O4	0.0534 (12)	0.0416 (12)	0.0440 (12)	0.0014 (9)	0.0062 (9)	0.0006 (9)
C1	0.0412 (15)	0.0398 (16)	0.0409 (16)	0.0028 (12)	0.0037 (12)	0.0013 (13)
C2	0.0448 (15)	0.0470 (17)	0.0362 (16)	0.0024 (13)	0.0034 (12)	0.0048 (13)
C3	0.0465 (16)	0.0443 (17)	0.0449 (17)	0.0009 (13)	0.0060 (13)	0.0093 (13)
C4	0.0378 (14)	0.0431 (16)	0.0410 (16)	0.0015 (12)	0.0025 (12)	0.0055 (13)
C5	0.0368 (14)	0.0465 (17)	0.0357 (16)	0.0038 (12)	0.0005 (11)	0.0016 (12)
C6	0.0366 (14)	0.0433 (17)	0.0407 (16)	0.0020 (12)	0.0002 (12)	0.0054 (13)
C7	0.0474 (16)	0.0479 (18)	0.0393 (16)	0.0001 (13)	0.0063 (12)	0.0037 (13)
C8	0.0488 (16)	0.0446 (17)	0.0432 (17)	0.0023 (13)	0.0078 (13)	0.0068 (14)
C9	0.0605 (19)	0.0501 (18)	0.0438 (18)	−0.0023 (15)	0.0129 (14)	−0.0003 (14)
C10	0.0493 (17)	0.0476 (17)	0.0427 (17)	0.0017 (13)	0.0057 (13)	0.0022 (14)
C11	0.0465 (16)	0.0529 (18)	0.0430 (17)	0.0071 (14)	0.0022 (13)	0.0035 (14)
C12	0.0529 (17)	0.0502 (18)	0.0427 (17)	0.0005 (14)	0.0035 (13)	−0.0057 (14)
C13	0.072 (2)	0.080 (3)	0.066 (2)	0.026 (2)	−0.0107 (18)	−0.019 (2)
C14	0.081 (3)	0.094 (3)	0.081 (3)	0.027 (2)	−0.007 (2)	−0.038 (2)
C15	0.080 (2)	0.081 (3)	0.045 (2)	0.003 (2)	0.0019 (17)	−0.0092 (18)
C16	0.062 (2)	0.061 (2)	0.0470 (19)	0.0081 (16)	0.0018 (15)	−0.0014 (15)
C17	0.0452 (16)	0.0491 (18)	0.0457 (17)	−0.0031 (14)	−0.0022 (13)	0.0055 (14)
C18	0.0489 (16)	0.0412 (17)	0.0443 (17)	0.0021 (13)	−0.0028 (13)	0.0046 (13)
C19	0.0511 (18)	0.0520 (19)	0.059 (2)	−0.0061 (15)	−0.0065 (15)	−0.0002 (16)
C20	0.080 (2)	0.051 (2)	0.057 (2)	−0.0020 (18)	−0.0036 (18)	−0.0091 (16)
C21	0.080 (2)	0.067 (2)	0.057 (2)	0.0141 (19)	0.0093 (18)	−0.0092 (18)
C22	0.0550 (19)	0.061 (2)	0.073 (2)	0.0063 (16)	0.0108 (16)	0.0037 (18)
C23	0.0523 (17)	0.0480 (18)	0.0551 (19)	0.0005 (14)	0.0007 (14)	0.0027 (15)
C24	0.0467 (16)	0.0507 (18)	0.0398 (17)	−0.0002 (13)	0.0014 (13)	0.0068 (14)
C25	0.0482 (16)	0.0480 (17)	0.0388 (16)	−0.0016 (13)	0.0094 (13)	0.0067 (13)
C26	0.0605 (19)	0.0560 (19)	0.0406 (17)	0.0045 (15)	0.0101 (14)	0.0016 (14)
C27	0.074 (2)	0.070 (2)	0.062 (2)	0.0198 (19)	0.0130 (17)	0.0144 (19)
C28	0.092 (3)	0.062 (2)	0.068 (3)	0.010 (2)	0.030 (2)	−0.0012 (19)
C29	0.082 (3)	0.076 (3)	0.050 (2)	0.005 (2)	0.0135 (19)	−0.0058 (19)
C30	0.064 (2)	0.066 (2)	0.0414 (18)	0.0011 (17)	0.0065 (15)	0.0019 (15)
C31	0.085 (2)	0.050 (2)	0.062 (2)	−0.0033 (17)	0.0115 (18)	−0.0044 (17)

### Geometric parameters (Å, º)

**Table:**

N1—C11	1.366 (4)	C13—C14	1.391 (6)
N1—N2	1.369 (4)	C13—H13	0.9300
N1—C12	1.437 (5)	C14—H14	0.9300
N2—N3	1.320 (4)	C15—C16	1.388 (5)
N3—C10	1.383 (4)	C15—H15	0.9300
N4—C15	1.337 (5)	C16—H16	0.9300
N4—C14	1.343 (6)	C17—C18	1.509 (5)
O1—C5	1.376 (4)	C18—C23	1.397 (5)
O1—C9	1.458 (4)	C18—C19	1.410 (5)
O2—C17	1.236 (4)	C19—C20	1.398 (5)
O3—C24	1.232 (4)	C19—H19	0.9300
O4—C1	1.378 (4)	C20—C21	1.392 (6)
O4—C8	1.412 (4)	C20—H20	0.9300
C1—C2	1.395 (4)	C21—C22	1.393 (5)
C1—C6	1.412 (5)	C21—H21	0.9300
C2—C3	1.398 (5)	C22—C23	1.401 (5)
C2—C24	1.505 (5)	C22—H22	0.9300
C3—C4	1.416 (5)	C23—H23	0.9300
C3—H3	0.9300	C24—C25	1.503 (5)
C4—C5	1.416 (4)	C25—C30	1.402 (5)
C4—C17	1.507 (5)	C25—C26	1.405 (5)
C5—C6	1.426 (5)	C26—C27	1.401 (5)
C6—C7	1.459 (5)	C26—H26	0.9300
C7—C8	1.352 (4)	C27—C28	1.389 (6)
C7—H7	0.9300	C27—H27	0.9300
C8—C31	1.480 (5)	C28—C29	1.399 (6)
C9—C10	1.497 (4)	C28—H28	0.9300
C9—H9A	0.9700	C29—C30	1.382 (5)
C9—H9B	0.9700	C29—H29	0.9300
C10—C11	1.369 (5)	C30—H30	0.9300
C11—H11	0.9300	C31—H31A	0.9600
C12—C16	1.376 (5)	C31—H31B	0.9600
C12—C13	1.402 (5)	C31—H31C	0.9600
			
C11—N1—N2	110.2 (3)	N4—C15—H15	117.3
C11—N1—C12	130.0 (3)	C16—C15—H15	117.3
N2—N1—C12	119.7 (3)	C12—C16—C15	118.3 (3)
N3—N2—N1	106.9 (3)	C12—C16—H16	120.8
N2—N3—C10	109.4 (3)	C15—C16—H16	120.8
C15—N4—C14	115.0 (3)	O2—C17—C4	117.9 (3)
C5—O1—C9	118.3 (2)	O2—C17—C18	118.5 (3)
C1—O4—C8	106.1 (2)	C4—C17—C18	123.5 (3)
O4—C1—C2	123.9 (3)	C23—C18—C19	118.8 (3)
O4—C1—C6	110.6 (2)	C23—C18—C17	123.1 (3)
C2—C1—C6	125.5 (3)	C19—C18—C17	117.8 (3)
C1—C2—C3	114.7 (3)	C20—C19—C18	120.4 (3)
C1—C2—C24	124.6 (3)	C20—C19—H19	119.8
C3—C2—C24	120.7 (3)	C18—C19—H19	119.8
C2—C3—C4	123.5 (3)	C21—C20—C19	120.3 (3)
C2—C3—H3	118.2	C21—C20—H20	119.8
C4—C3—H3	118.2	C19—C20—H20	119.8
C5—C4—C3	119.9 (3)	C22—C21—C20	119.7 (3)
C5—C4—C17	125.1 (3)	C22—C21—H21	120.2
C3—C4—C17	114.9 (2)	C20—C21—H21	120.2
O1—C5—C4	117.3 (3)	C21—C22—C23	120.3 (3)
O1—C5—C6	124.3 (3)	C21—C22—H22	119.8
C4—C5—C6	118.3 (3)	C23—C22—H22	119.8
C1—C6—C5	118.1 (3)	C18—C23—C22	120.5 (3)
C1—C6—C7	104.9 (3)	C18—C23—H23	119.7
C5—C6—C7	136.9 (3)	C22—C23—H23	119.7
C8—C7—C6	107.4 (3)	O3—C24—C25	120.7 (3)
C8—C7—H7	126.3	O3—C24—C2	120.1 (3)
C6—C7—H7	126.3	C25—C24—C2	119.3 (3)
C7—C8—O4	110.9 (3)	C30—C25—C26	118.7 (3)
C7—C8—C31	133.4 (3)	C30—C25—C24	119.5 (3)
O4—C8—C31	115.6 (3)	C26—C25—C24	121.7 (3)
O1—C9—C10	109.8 (3)	C27—C26—C25	120.2 (3)
O1—C9—H9A	109.7	C27—C26—H26	119.9
C10—C9—H9A	109.7	C25—C26—H26	119.9
O1—C9—H9B	109.7	C28—C27—C26	120.1 (3)
C10—C9—H9B	109.7	C28—C27—H27	120.0
H9A—C9—H9B	108.2	C26—C27—H27	120.0
C11—C10—N3	107.9 (3)	C27—C28—C29	120.1 (4)
C11—C10—C9	131.1 (3)	C27—C28—H28	120.0
N3—C10—C9	121.0 (3)	C29—C28—H28	120.0
N1—C11—C10	105.6 (3)	C30—C29—C28	119.8 (3)
N1—C11—H11	127.2	C30—C29—H29	120.1
C10—C11—H11	127.2	C28—C29—H29	120.1
C16—C12—C13	118.5 (3)	C29—C30—C25	121.2 (3)
C16—C12—N1	121.7 (3)	C29—C30—H30	119.4
C13—C12—N1	119.8 (3)	C25—C30—H30	119.4
C14—C13—C12	117.9 (3)	C8—C31—H31A	109.5
C14—C13—H13	121.0	C8—C31—H31B	109.5
C12—C13—H13	121.0	H31A—C31—H31B	109.5
N4—C14—C13	124.9 (4)	C8—C31—H31C	109.5
N4—C14—H14	117.6	H31A—C31—H31C	109.5
C13—C14—H14	117.6	H31B—C31—H31C	109.5
N4—C15—C16	125.4 (4)		
			
C11—N1—N2—N3	0.5 (3)	N2—N1—C12—C16	−168.3 (3)
C12—N1—N2—N3	177.1 (3)	C11—N1—C12—C13	−172.8 (3)
N1—N2—N3—C10	0.3 (4)	N2—N1—C12—C13	11.3 (4)
C8—O4—C1—C2	177.6 (2)	C16—C12—C13—C14	0.5 (5)
C8—O4—C1—C6	−0.1 (3)	N1—C12—C13—C14	−179.0 (3)
O4—C1—C2—C3	−177.4 (2)	C15—N4—C14—C13	0.0 (6)
C6—C1—C2—C3	0.0 (4)	C12—C13—C14—N4	−0.5 (7)
O4—C1—C2—C24	−1.4 (4)	C14—N4—C15—C16	0.6 (6)
C6—C1—C2—C24	176.0 (3)	C13—C12—C16—C15	−0.1 (5)
C1—C2—C3—C4	0.5 (4)	N1—C12—C16—C15	179.5 (3)
C24—C2—C3—C4	−175.7 (2)	N4—C15—C16—C12	−0.5 (6)
C2—C3—C4—C5	0.3 (4)	C5—C4—C17—O2	−144.0 (3)
C2—C3—C4—C17	−176.2 (2)	C3—C4—C17—O2	32.3 (4)
C9—O1—C5—C4	−158.5 (2)	C5—C4—C17—C18	41.3 (4)
C9—O1—C5—C6	23.3 (4)	C3—C4—C17—C18	−142.4 (3)
C3—C4—C5—O1	−179.9 (2)	O2—C17—C18—C23	−149.1 (3)
C17—C4—C5—O1	−3.7 (4)	C4—C17—C18—C23	25.6 (4)
C3—C4—C5—C6	−1.6 (4)	O2—C17—C18—C19	23.6 (4)
C17—C4—C5—C6	174.6 (2)	C4—C17—C18—C19	−161.7 (3)
O4—C1—C6—C5	176.4 (2)	C23—C18—C19—C20	0.4 (5)
C2—C1—C6—C5	−1.3 (4)	C17—C18—C19—C20	−172.6 (3)
O4—C1—C6—C7	−1.1 (3)	C18—C19—C20—C21	0.1 (5)
C2—C1—C6—C7	−178.7 (3)	C19—C20—C21—C22	−0.3 (5)
O1—C5—C6—C1	−179.8 (2)	C20—C21—C22—C23	0.0 (5)
C4—C5—C6—C1	2.0 (4)	C19—C18—C23—C22	−0.7 (5)
O1—C5—C6—C7	−3.4 (5)	C17—C18—C23—C22	171.9 (3)
C4—C5—C6—C7	178.4 (3)	C21—C22—C23—C18	0.5 (5)
C1—C6—C7—C8	1.9 (3)	C1—C2—C24—O3	−128.6 (3)
C5—C6—C7—C8	−174.9 (3)	C3—C2—C24—O3	47.1 (4)
C6—C7—C8—O4	−2.0 (3)	C1—C2—C24—C25	52.4 (4)
C6—C7—C8—C31	176.2 (3)	C3—C2—C24—C25	−131.8 (3)
C1—O4—C8—C7	1.3 (3)	O3—C24—C25—C30	20.2 (4)
C1—O4—C8—C31	−177.2 (2)	C2—C24—C25—C30	−160.8 (3)
C5—O1—C9—C10	149.1 (2)	O3—C24—C25—C26	−155.7 (3)
N2—N3—C10—C11	−0.9 (4)	C2—C24—C25—C26	23.2 (4)
N2—N3—C10—C9	−177.8 (3)	C30—C25—C26—C27	−1.2 (4)
O1—C9—C10—C11	93.6 (4)	C24—C25—C26—C27	174.8 (3)
O1—C9—C10—N3	−90.4 (4)	C25—C26—C27—C28	1.8 (5)
N2—N1—C11—C10	−1.0 (3)	C26—C27—C28—C29	−0.5 (5)
C12—N1—C11—C10	−177.2 (3)	C27—C28—C29—C30	−1.2 (6)
N3—C10—C11—N1	1.2 (3)	C28—C29—C30—C25	1.8 (5)
C9—C10—C11—N1	177.6 (3)	C26—C25—C30—C29	−0.5 (5)
C11—N1—C12—C16	7.6 (5)	C24—C25—C30—C29	−176.6 (3)

### Hydrogen-bond geometry (Å, º)

**Table:**

*D*—H···*A*	*D*—H	H···*A*	*D*···*A*	*D*—H···*A*
C11—H11···O3^i^	0.93	2.33	3.225 (10)	161
C16—H16···O3^i^	0.93	2.55	3.473 (10)	171

Symmetry code: (i) *x*, *y*, *z*−1.
